# Valued technical and non-technical skills among disaster responders: a cross sectional study of disaster responders involved in the earthquake in Türkiye and Syria January 2023

**DOI:** 10.1186/s12873-024-01083-x

**Published:** 2024-09-23

**Authors:** Anja Westman, Lisa Kurland, Karin Hugelius

**Affiliations:** 1 Faculty of Medicine and Health, Örebro University, Örebro, Sweden; 2 Örebro University Hospital, Region Örebro, Örebro, Sweden

## Abstract

**Introduction:**

Disaster responders are an important part of disaster response. However, despite large efforts to train disaster responders, there is a limited scientific knowledge regarding which competences and skills such responders value and lack during a real mission. The aim of this study was to investigate used and needed skills among disaster responders responding to the earthquake in Türkiye and Syria 2023

**Methods:**

A cross-sectional study using a non-randomized sample was conducted, collected between March and July, 2023. The participants were recruited through invitations distributed to international organizations, and the data were gathered through a web-based survey. The data were analyzed using descriptive and comparative statistics.

**Results:**

A total of 525 participants involved in the disaster response in Türkiye or Syria in February 2023 were included. The most common valued skills were teamwork skills (*n* = 252, 59%), technical knowledge (*n* = 204, 48%), leadership skills (*n* = 105, 24%) and communication skills (*n* = 114, 17%). Women valued stress management (*n* = 33, 26%) more than leadership (*n* = 24, 19%) Technical knowledges were more valued among first-time responders (*n* = 168, 82%) compared to experienced responders (*n* = 108, 54%, p-value < 0.001). The most reported lacked skills were mental preparedness (*n* = 237, 53%), knowledge of the management system of international response (*n* = 132, 30%), stress management (*n* = 105, 24%) and leadership (*n* = 102, 23%).

**Conclusion:**

The results showed slightly different needs in the various phases of a response, as well as some differences between men and women. Improving mental preparedness was not one of the most highly valued skills, but it was one of the skills that was most lacking; this discrepancy is an interesting finding. More in-depth analysis and additional studies are needed to further understand how best to prepare disaster responders and how their training can include the desirable skills. Further studies should be focused on the experience and knowledge of qualified disaster responders. This knowledge could also be of use when recruiting since several of the non-technical skills are not only gained solely through specific training.

**Supplementary Information:**

The online version contains supplementary material available at 10.1186/s12873-024-01083-x.

## Background

Disasters and natural hazards strike worldwide and often with devastating impact on the societies where they occur. According to annual report of the Emergency Event Database (EM-DAT) 399 events were reported in 2023 [1, 2]. Birnbaum’s conceptual framework describes how hazards evolve into events that causes structural (e.g., human injuries or damaged infrastructure) and functional damage (e.g., loss of essential societal functions, such as overwhelmed healthcare services) [3]. The structural and functional damage creates needs that lead to an emergency or disaster which requires a response in the form of interventions or actions undertaken to meet these needs. Extraordinary responses include additional backup equipment, services, and personnel. If the ordinary and extraordinary response capacity is not sufficient to meet the needs, outside response capacity is needed [3]. The affected community’s ability to manage the needs resulting from the functional damage caused by the event determine whether a disaster occurs. A simplified definition is that if outside assistance is needed to meet the needs, a disaster has occurred.

Disaster response has been described as “actions taken directly before, during or immediately after a disaster in order to save lives, reduce health impacts, ensure public safety and meet the basic subsistence needs of the people affected” [4]. In the current study, disaster responders are defined as individuals responding to a disaster as part of their ordinary duty or a specific deployment who are an important part of the disaster response. The nature of disasters comprises safety and security risks, high levels of uncertainty, unclear organizational structures, different working methods, and the ability to face human suffering [5]. Therefore, responding to disasters require specific competences, skills, and preparedness of both individual disaster responders and response teams [6]. However, there is still no consensus on which competences that are essential for disaster responders [7].

Skills are a type of competence and can be divided in non-technical skills (NTS) and technical skills (TS). NTS are skills of an individual and/or team that combines cognitive and social components; they are necessary for avoiding error and improving individual and/or team performance [8]. Although NTS such as communication and teamwork are recognized, for example, in the aviation industry and slowly becoming more important in some areas of healthcare, their use within disaster response is not yet standard [9]. Previous research indicates that NTS are of value alongside with TS but little information is available on which NTS are the most important. Many studies are also based on training environment and/or use a relatively small study sample [10].

The deadliest disaster of 2023 occurred in the morning of February 6, when a 7.8-magnitude earthquake struck the border area between Türkiye and northern Syria, (GLIDE EQ-2023-000015-TUR and EQ-2023-000015-SYR) [11]. It was followed by hundreds of smaller aftershocks and another powerful earthquake. The estimated number of damaged buildings is over 500,000 and the mortality rate exceeded 50,000 people. The enormous impact of the event on the area lead to large national disaster response and a massive international one [12]. Urban search and rescue (USAR) teams from 83 countries, comprising approximately 10,000 personnel and more than 300 search dogs, were deployed to the area as well as national and international emergency medical teams (EMT), humanitarian experts and organizations [13].

To deepen the understanding of how to prepare disaster responders, this study aims to investigate which competences and skills disaster responders perceived as valuable and which they lacked while responding to the earthquake in Türkiye and Syria 2023. The study also aims to analyze differences of perceived valuable and lacking competences in relation to sex, previous experience, nationality, and length of mission. This information could then be of use when recruiting and training disaster responders.

## Methods

### Design

A cross-sectional study using a non-randomized sample was conducted.

Participants:

Eligible participants were disaster responders who were involved in the disaster response in Türkiye/Syria in February 2023. The participants were required to be above 18 years of age and able to answer the survey in English or Turkish. No exclusion criteria was used.

### Data Collection procedures

The data were gathered from a web-based survey, developed specifically for the current study, based on previous studies [10, 14]. The multiple-choice questionnaire encompassed questions on which skill (TS and several NTS) the responders considered most important and which skills (TS and several NTS) the responders lacked. In the survey, technical knowledge was described as “how to do rescue in rubble, medical procedures or information management.” Additionally, knowledge of the management system for international response and coordination was considered and specified as a TS. The NTS specified in the survey were communication skills, teamwork skills, leadership skills, task management skills, decision-making skills, stress management, professional networks, and mental preparedness improvement. The questions that were asked and analyzed in this study are presented in Appendix 1.

The questionnaire was available in English and Turkish and was piloted among ten Swedish aid workers with experience as disaster responders. The questionnaire also requested data as age, sex, profession, and previous experience.

The invitation to participate was distributed by international organizations, including the International Federation of the Red Cross, Médecins Sans Frontières, Save the Children, United Nations Office for the Coordination of Humanitarian Affairs, World Food Program, United Nations Children’s Fund, the World Health Organization’s EMT Secretariat, and the International Search and Rescue Advisory Group, as well as several non-governmental organizations. The invitation was sent out eight weeks after the earthquake and was open from March 24 to July 30, 2023.

### Data analysis

The data were analyzed using both descriptive statistics (mean, median, and percentages) and comparative statistics for comparisons between sex (men and women), length of mission (mission ≤ 7 days and ≥ 8 days), and type of responders (national or international). IBM SPSS Statistics, version 29.0.2.0 (20) was used. Additionally, a Chi2-test correlated with the Benjamini-Hochberg method was used, and a statistician was consulted during the analysis process. A p-value < 0.05 were considered significant.

### Ethical considerations

The study was approved by the Swedish Ethical Review board (reference number 2023-01369-02) and was conducted in line with the standards of good research ethics. [15]. All data were collected and analyzed anonymized and informed consent was obtained as part of the survey.

## Results

The survey was answered by 525 disaster responders: 165 (31%) women and 360 (69%) men. The mean age of participants was 42 years (median 40, SD 10,5), with a range of 22 to 65. Of the responders 102 (19%) were national, while 423 (81%) were international. Their most common positions during the mission were as USAR (*n* = 123, 23%), EMT (*n* = 108, 21%) or within management coordination (*n* = 87, 17%). Of the 525 disaster responders 204 (39%) had been on at least one prior mission, while 288 (55%) responders were on their first mission (35 missing answers).

Previous experience ranged from 0 to 15 missions, and 18 (3%) disaster responders had ten or more previous missions. The length of stay within the mission varied. 246 (47%) of the disaster responders stayed for up to seven days. See Table 1.

**Table 1:** Descriptive data of the participants

		**Frequency n (%)**					**Frequency n (%)**			
Total study population *N* = 525										
**Sex**	Women	165 (31)	**Position**	USAR			123 (23)			
	Men	360 (69)		EMT			108 (21)			
**Age**	Min-max	22–65		Management coordination			87 (17)			
	Mean (median) Standard deviation	42 (40) 10,5		Health/medical			48 (9)			
**Length of mission**	1–7 days	246 (47)		Other humanitarian aid			45 (9)			
	8–21 days > 21 days	186 (35) 93 (18)		Mental health and psychosocial support			36 (7)			
**No of missions**	1 (including current)	288 (55)		Needs assessment			24 (5)			
	2–9	186 (35)		Food or nutrition			21 (4)			
	10–15	18 (3)		Shelter			18 (3)			
**Nationality**	National	102 (19)		Water and sanitation			9 (2)			
	International	423 (81)		Early recovery			3 (0.6)			

### Skills perceived as most valuable

The skills reported as being most important for the ability to act efficiently in regard to the disaster response were technical knowledge (*n* = 297, 69%), teamwork skills (*n* = 252, 59%), knowledge of the system for international response and coordination (*n* = 204, 48%), leadership skills (*n* = 105, 24%), and communication skills (*n* = 114, 17%). Women valued stress management (*n* = 33, 26%) as more highly than leadership skills (*n* = 24, 19%).

Table 2. Valued skills in relation to sex, previous experience, nationality, and length of mission.

**Table 2:** Valued skills in relation to sex, previous experience, national or international responder and length of mission

		Sex					Experience						Type of responder									Length of mission								
Valued skills*		Total study sample *N* = 429 n (%)	Women (*n* = 129) n (%)	Men (*n* = 300) n (%)	*p*-value **	Adjusted *p* ***	First time responder (*n* = 204) n (%)	Experienced responders (*n* = 201) n (%)	*p*-value **		Adjusted *p* ***			National staff (*n* = 42) n (%)		International staff (*n* = 387) n (%)		*p*-value **		Adjusted *p* ***			≤ 7 days (*n* = 186) n (%)		≥ 8 days (*n* = 243) n (%)		*p*-value **		Adjusted *p* ***,	
Technical skills	Technical knowledge	297 (69)	72 (56)	225 (75)	< 0.001	0.004	168 (82)	108 (54)	< 0.001	0.004		21 (50)			276 (71)		0.008		0.023		153 (82)			144 (60)		< 0.001		0.004		
	Knowledge on the international response system	204 (48)	39 (30)	165 (55)	< 0.001	0.004	75 (37)	117 (58)	< 0.001	0.004		15 (36)			99 (26)		0.197		0.300		69 (37)			135 (56)		< 0.001		0.004		
Non- technical skills	Communication skills	114 (27)	30 (23)	84 (28)	0.341	0.411	45 (22)	60 (30)	0.089	0.150		15 (36)			90 (23)		0.089		0.150		45 (24)			69 (28)		0.378		0.443		
	Leadership skills	105 (25)	24 (19)	81 (27)	0.067	0.123	39 (19)	57 (28)	0.035	0.076		9 (21)			33 (9)		0.013		0.036		42(23)			63(26)		0.497		0.55		
	Decision making	42 (10)	12 (9)	30 (10)	1.000	1.000	18 (9)	24 (12)	0.331	0.411		9 (21)			36 (9)		0.029		0.07		6(3)			36(15)		< 0.001		0.004		
	Professional network	45 (11)	15 (12)	30 (10)	0.609	0.660	21 (10)	21 (10)	1.000	1.000		12 (29)			192 (50)		0.014		0.036		15(8)			30(12)		0.203		0.300		
	Teamwork skills	252 (59)	81 (63)	171 (57)	0.286	0.370	111 (54)	129 (64)	0.055	0.113		21 (50)			231 (60)		0.250		0.350		93(50)			159(65)		0.002		0.007		
	Task management	24 (6)	3 (2)	21 (7)	0.066	0,125	15 (7)	9 (5)	0.293	0.380		6 (14)			18 (5)		0.022		0.053		6 (3)			18 (7)		0.088		0.146		
	Stress management	69 (16)	33 (26)	36 (12)	< 0.001	0.004	27 (13)	39 (19)	0.107	0.170		18 (43)			51 (13)		< 0.001		0.004		18 (10)			51 (21)		0.001		0.004		
	Building mental preparedness	60 (14)	15 (12)	45 (15)	0.448	0.510	33 (16)	24 (12)	0.254	0.350		12 (29)			48 (12)		0.008		0.023		12 (7)			48 (20)		< 0.001		0.004		

* Total *N* = 527, valid answers *N* = 429 (missing *n* = 98). ** Chi 2 test *** Benjamini-Hochberg test

Teamwork skills were valued highly by both men and women − 60% and 57%, respectively - as well as by first-time responders (*n* = 111, 54%) and experienced responders (*n* = 129, 64%, *p =* 0.055). A difference was seen when compared to the length of the mission. Of the responders, 50% with missions shorter than 7 days (*n* = 93) valued teamwork, compared to 65% of responders with longer missions (*n* = 159, *p =* 0.002).

For *technical knowledge*, there was a difference between the sexes: 75% (*n* = 225) of the men valued this skill highly compared to 56% (*n* = 72, *p <* 0.001) of the women. It was also valued more highly among first-time responders (*n* = 168, 82%) than experienced responders (*n* = 108, 54%, *p* < 0.001). National disaster responders (*n* = 21, 50%) considered technical knowledge less important than their international counterparts (*n* = 276, 71%, *p* = 0.008). There was a significant difference between responders with shorter missions (≤ 7 days, *n* = 153, 82%) compared to those with longer missions (≥ 8 days, *n* = 144, 60%, *p* < 0.001).

Knowledge of the system for international response and coordination was valued as important by 48% of the disaster responders, and 55% (*n* = 165) of the men valued the skill highly compared to 30% of the women (*n* = 39, *p* < 0.001). It was valued more highly among national (*n* = 15, 36%) than international responders (*n* = 99, 26%), although the difference was not significant. Knowledge of system management was valued more highly among those with missions longer than 8 days (*n* = 135, 56%) compared to those with missions shorter than a week (*n* = 69, 37%, *p* < 0.001). See Table 2.

Leadership was valued more highly by men (27%, *n* = 81) than women (19%, *n* = 24). No significant changes could be seen over time; however, the experienced disaster responders (*n* = 57, 28%) valued leadership skills as somewhat more important than first-time disaster responders (*n* = 39, 19%, *p =* 0.035).

Communication was valued by men and women as one of the top three NTS (27% of all responders), and there was minimal difference between the sexes (28%, *n* = 84 of the men and 23%, *n* = 30 of the women). It was valued slightly more highly among experienced (*n* = 60, 30%) than first-time responders (*n* = 45, 22%, *p =* 0.089). National responders (*n* = 15, 36%) valued communication slightly more highly than international responders (*n* = 99, 23%), and its importance increased with length of mission. Of the responders with missions shorter than 7 days, 24% (*n* = 45) valued communication highly compared to 28% (*n* = 69) for disaster responders with missions over 8 days, however this difference was not statistically significant.

### Skills perceived as lacking

The most commonly reported lacking skills were mental preparedness improvement (*n* = 237, 53%), knowledge of the system for international response and coordination (*n* = 132, 30%), stress management (*n* = 105, 24%), leadership (*n* = 102, 23%), and communication (*n* = 96, 22%).

Table 3. Lacked skills in relation to sex, previous experience, nationality, and length of mission.

**Table 3:** Lacked skills in relation to sex, previous experience, national or international responder and length of mission

		Sex					Experience				Type of responder				Length of mission			
Lacked skills*		Total study sample *N* = 444 (%)	Women (*n* = 141) (%)	Men (*n* = 303) (%)	*p*-value **	Adjusted *p* ***	First time responders (*n* = 273) n (%)	Experiences responders (*n* = 141) n (%)	*p*-value **	Adjusted *p* ***	National staff (*n* = 93) n (%)	International (*n* = 351) n (%)	*p*-value **	Adjusted *p* ***	≤ 7 days (*n* = 222) n (%)	≥ 8 days (*n* = 222) n (%)	*p*-value **	Adjusted *p* ***
Technical skills	Technical knowledge	54 (12)	12 (9)	42 (14)	0.120	0.200	36 (13)	9 (6)	0.045	0.100	27 (29)	27 (8)	< 0.001	0.005	33(15)	21 (10)	0.110	0.200
	Knowledge on the international response system	132 (30)	42 (30)	90 (30)	1.000	1.000	102 (37)	27 (19)	< 0.001	0.005	18 (19)	78 (22)	0.671	0.809	84 (38)	48 (22)	< 0.001	0.005
Non- technical skills	Communication skills	96 (22)	30 (21)	66 (22)	1.000	1.000	57 (21)	33 (23)	0.615	0.764	18 (19)	84 (24)	0.407	0.600	45 (20)	51 (23)	0.564	0.754
	Leadership skills	102 (23)	39 (28)	63 (21)	0.116	0.200	63 (23)	33 (23)	1.000	1.000	12 (13)	39 (11)	0.589	0.800	39 (18)	63 (28)	0.009	0.023
	Decision making	51 (12)	12 (9)	39 (13)	0.203	0.320	21 (8)	24 (17)	0.007	0.020	9 (10)	60 (17)	0.106	0.200	6 (3)	45 (20)	< 0.001	0.005
	Professional network	69 (16)	15 (11)	54 (18)	0.067	0.140	42 (15)	21 (15)	1.000	1.000	36 (39)	96 (27)	0.041	0.093	27 (12)	42 (19)	0.006	0.019
	Teamwork skills	69 (16)	24 (17)	45 (15)	0.575	0.754	54 (20)	9 (6)	< 0.001	0.005	24 (26)	45 (13)	0.004	0.015	45 (20)	24 (11)	0.008	0.022
	Task management	30 (7)	9 (30)	21 (70)	1.000	1.000	15 (6)	12 (9)	0.293	0.444	12 (13)	18 (5)	0.017	0.041	3 (1)	27 (12)	< 0.001	0.005
	Stress management	105 (24)	21 (15)	84 (28)	0.003	0.012	57 (21)	32 (26)	0.320	0.470	12 (13)	93 (27)	0.006	0.019	45 (20)	60 (27)	0.118	0.200
	Building mental preparedness	237 (53)	75 (53)	162 (54)	1.000	1.000	174 (64)	51 (36)	< 0.001	0.005	36 (39)	201 (57)	0.002	0.009	138 (62)	99 (45)	< 0.001	0.005

* Total *N* = 527, valid answers *N* = 444 (missing *n* = 83). ** Chi 2 test *** Benjamini-Hochberg test

Knowledge of how to improve mental preparedness was not valued as one of the most important skills but was a commonly lacked skill. Both men (54%) and women (53%) reported that they lacked this skill. Of the national responders, 39% (*n* = 36) lacked sufficient mental preparedness, and for the international responders, this figure was 57% (*n* = 201, p 0.002). However, fewer of those with missions over 8 days reported a lack of mental preparedness (*n* = 99, 45%) compared to responders with short missions (≤ 7 days) (*n* = 138, 62%, *p* < 0.001).

Knowledge of the system for international response and coordination was reported as a lacking skill by first-time responders compared to experienced disaster responders to a significant level, (37% (*n* = 102), 19% (*n* = 19), *p* < 0.001). Similarly, there was a significant difference between the responders on shorter missions (≤ 7 days *n* = 84, 38%) and those on longer missions (≥ 8 days *n* = 48, 22%, *p* < 0.001). However, there were no significant differences between international or national responders or between the sexes.

Lack of *stress management skills* was reported to a higher level by men (*n* = 84, 28%) compared to women (*n* = 21, 15%, *p* 0.003). Additionally, it was reported more frequently among international (*n* = 93, 27%) than national responders (*n* = 12, 13%, *p* 0.002), but no significant difference regarding length of mission was noted.

Both men and women reported lacking *leadership skills*, and there was no significant difference between first-time responders (*n* = 63, 23%) and those with experience (*n* = 33, 23%). National (*n* = 18, 19%) and international (*n* = 84, 24%) disaster responders both reported that the skill was severely lacking.

Regarding the perceived lack of *communication skills*, there was no significant difference between men (*n* = 66, 22%) and women (*n* = 30, 21%) nor in relation to previous experience, type of responders, or length of mission.

## Discussion

The disaster responders in this study perceived both TS and NTS as important for their ability to respond effectively to the earthquake. The most highly valued skills were technical knowledge, knowledge of the system for international response and coordination, teamwork, leadership, and communication. Among the perceived lacking skills, mental preparedness, knowledge of the system for international response and coordination, stress management, and leadership were most often reported. Sex, length of mission, type of responder, and previous experience had an influence on the reported valued and lacking skills.

The results show that perceived valued skills and perceived lacking skills varied depending on length of mission as well as between men and women. However, these differences could also depend on the disaster response phases or activities. For example, USAR team members were mainly men, and the USAR teams were deployed in the immediate aftermath of the earthquake and with a short mission. Despite this, the significant differences in skills perceived as valued or lacking in relation to mission length are interesting. The result implies that teamwork and stress management as well as skills on how to build and improve mental health became more important for responders with longer missions. Improving mental preparedness was not one of the highest valued skills but perceived as one of the most lacing; this discrepancy is another interesting finding.

Mental preparedness, along with other NTS, tends to be given less attention than TS and training in these skills [16]. The results of the current study support the need for more training in mental preparedness and stress management. Factors affecting how to cope are of importance in a disaster setting but do not differ from factors in other field wherein it is known that good leadership, functional organization, and work culture play significant roles [17].

The participants in the current study consisted of both international and national disaster responders. In all disasters, it is the first responders –that is, the local healthcare or rescue service– that have the largest impact [18]. However, in a disastrous event, when local and national capacities are overwhelmed, international response is often needed at some point. International disaster responders may have different needs from local or regional teams; this is supported by the results of the current study. International teams are an essential part of disaster response [19, 20], but assessment of the effectiveness of such teams is insufficient, and research is lacking. Standardization of competency set, or framework is also lacking, resulting in inconsistency of functionality [3, 7]. From an organizational and minimal standard perspective, this development of framework regarding teams such as USAR and EMT is important [21]. The current study contributes by adding knowledge regarding which skills disaster responders value more than others and could therefore become useful when selecting responders for futural missions.

NTS are gaining increasing attention within the medical field owing to their impact on both educational environments and clinical practice [22, 23]. In disaster medicine, NTS are crucial, but their effective integration into real-world settings remains a challenge [10]. In this study, two TS and three NTS were rated as most important. The result that NTS such as teamwork skills, leadership skills, and communication skills were valued highly is well in line with those of previous research [10, 24]. Experienced responders valued leadership skills, communication, and decision-making more highly than their inexperienced colleagues, perhaps suggesting that needs differ depending on previous experience and knowledge.

Disaster response requires prepared disaster responders. Every activity in relation to disaster preparedness, including training and education, enhance knowledge, skills, and attitudes towards disaster preparedness [25]. There is no common agreement on which competencies or skills are needed for disaster responders, and there are discrepancies in the curricula for training [21]. The current study indicates that the skills valued as important, and skills lacked vary over length of deployment. Therefore, further efforts must be made on to investigate core competences and skills needed for disaster responders and how such skills can be developed [24].

### Limitations

The current study has several limitations. One major limitation is that it relies on a non-randomized sample. Because there is no information on the number of responders being deployed nationally or internationally to this specific response, it is impossible to analyze the representativeness of the study sample. However, previous research in the field consists of small study samples (from 8 to 59) and/or training and simulated events [10]. The current study included 525 participants and can therefore be considered a sufficient number to enable one to draw reliable conclusions. Another limitation is that several variables were not collected, including when the responders were on site, their nationalities; and whether they had specific positions, such as team leader. Such information would have been valuable for conducting subgroup analysis. Some available demographic data (e.g. profession in ordinary workplace) collected was not used in the current study as the study focuses on disaster responders. The responder’s role during the mission rather than their ordinary work, was priority to describe.

It should be noted that the current study relies on a survey in which other information regarding the responder’s health and well-being was also gathered and will be presented elsewhere.

## Conclusion

Skills important for disaster responders include several TS and NTS, and the value of specific skills seem to vary in different phases of the response. The skills reported as most valuable were technical knowledge, teamwork skills, knowledge of the system for international response and coordination, leadership skills and communication skills. Among the skills perceived as lacking improvement of mental preparedness, knowledge of the system for international response and coordination, stress management, and leadership were reported most. Sex, previous experience, nationality, and length of mission all had an influence on the reported valuable and lacking skills. In addition to traditional NTS, mental preparedness is a critical component of disaster response readiness, which is also shown in the current study.

More detailed analysis and further studies are required to optimize training for disaster responders by incorporating these key skills. These findings can also be valuable for recruitment, as many NTS are acquired through experience rather than formal training.

## Electronic supplementary material

Below is the link to the electronic supplementary material.

Supplementary Material 1

## Data Availability

Availability of data and materialsThe data that support the findings of this study are not openly available due to reasons of sensitivity and are available from the corresponding author upon reasonable request.

## Abbreviations

EM: DAT-Emergency Event Database
EMT: Emergency Medical Teams
NTS: Non-Technical Skills
TS: Technical Skills
USAR: Urban Search And Rescue
