# Supplementary material for: Valued technical and non-technical skills among disaster responders: a cross sectional study of disaster responders involved in the earthquake in Türkiye and Syria January 2023
Source: BMC Emerg Med. 2024 Sep 23;24:171. doi: 10.1186/s12873-024-01083-x (PMC11421104; doi:10.1186/s12873-024-01083-x)
Supplement: Supplementary file 1 — Supplementary Material 1 [file 12873_2024_1083_MOESM1_ESM.docx]

Appendix 1. Survey questions.

Are you

- Female
- Male
- Other
- I don’t want to answer this question

How old are you?

What is you profession?

- Medical doctor/physician
- Nurse
- Medic/paramedic
- Rescuer/fireman
- Building engineer
- Police, military professional
- Social work/psychologist
- Logistic officer
- Information and Communication Technology Officer
- Water and sanitation expert
- Humanitarian aidworker
- Other (please comment below)

Did you participate in the response as

- Part of your ordinary work (for example as a nurse or firemen working every day in the disaster area)
- Local responder in a temporary deployment (for example as part of a local medical team deployed to the disaster area)
- International staff (for example as part of an international team)
- Other

Did you participate in the response as

- Part of your ordinary work, mandatory deployment
- Part of your ordinary work, voluntary deployment
- A temporary, mandatory deployment
- A temporary, voluntary deployment
- Other

How many international missions have you been deployed in (including this)?

What was your function/position during this mission?

- Emergency Medical Team (EMT)
- Health- or Medical (not EMT)
- Mental health and psychosocial support
- Urban Search and Rescue (USAR)
- Needs assessment
- Management, coordination and logistics (such as UNDAC or EUCPT)
- Shelter
- Food or nutrition
- Water and sanitation
- Early recovery
- Other humanitarian aid
- Other

How long was your deployment/mission?

- 1-7 days
- 8-21 days
- More than 21 days

If you recall trainings and exercises you underwent before this disaster, which topics or skills gained were most important for you ability to act in this situation? (Mark up to three alternatives).

- Technical knowledge within my field (such as how to rescue in rubble, medical procedures or information management)
- Management system for international response and coordination
- Communication skills
- Teamwork skills
- Leadership skills
- Task management skills
- Decision making skills
- Stress management
- Professional networks
- To build mental preparedness
- Other

Were there any valuable competencies or skills you lacked that would have been valuable for your ability to act in this situation? (Mark up to three alternatives)

- Technical knowledge within my field (such as how to do rescue in rubble, medical procedures or information management)
- Knowledge of the system for international response and coordination
- Communication skills
- Teamwork skills
- Leadership skills
- Task management skills
- Decision making skills
- Stress management
- Professional networks
- To build mental preparedness
- Other
